# Noise Levels Due to Commercial and Leisure Activities in Urban Areas: Experimental Validation of a Numerical Model Fed with Crowd Density Estimation Using Computer Vision

**DOI:** 10.3390/s25123604

**Published:** 2025-06-08

**Authors:** Óscar Ramón-Turner, Jacob D. R. Bordón, Asunción González-Rodríguez, Javier Lorenzo-Navarro, Modesto Castrillón-Santana, Guillermo M. Álamo, Román Quevedo-Reina, Carlos Romero-Sánchez, Antonio T. Ester-Sánchez, Cristina Medina, Fidel García, Orlando Maeso, Juan J. Aznárez

**Affiliations:** 1 Instituto Universitario de Sistemas Inteligentes y Aplicaciones Numéricas en Ingeniería, Universidad de Las Palmas de Gran Canaria, Campus de Tafira, 35017 Las Palmas, Spain; oscar.ramon@ulpgc.es (Ó.R.-T.); jacobdavid.rodriguezbordon@ulpgc.es (J.D.R.B.); asuncion.gonzalez@ulpgc.es (A.G.-R.); javier.lorenzo@ulpgc.es (J.L.-N.); modesto.castrillon@ulpgc.es (M.C.-S.); guillermo.alamo@ulpgc.es (G.M.Á.); roman.quevedo@ulpgc.es (R.Q.-R.); carlos.romero@ulpgc.es (C.R.-S.); cristina.medina@ulpgc.es (C.M.); fidel.garcia@ulpgc.es (F.G.); orlando.maeso@ulpgc.es (O.M.); 2 Facultad de Ciencias Jurídicas, Universidad de Las Palmas de Gran Canaria, Campus de Tafira, 35017 Las Palmas, Spain; tirso.ester@ulpgc.es

**Keywords:** urban noise, meshfree methods, noise prediction, leisure noise, noise sensors, crowd density, artificial neural networks, computer vision

## Abstract

Noise levels of anthropogenic origin in urban environments have reached thresholds that pose serious public health and quality of life problems. This paper/work aims to examine these noise levels, the underlying causes of their increase and possible solutions through the implementation of predictive models. To address this problem, as a first step, a simplified mathematical model capable of accurately predicting anthropogenic noise levels in a given area is developed. As variables, this model considers the crowd density, estimated using an Artificial Neural Network (ANN) capable of detecting people in images, as well as the geometric and architectural characteristics of the environment. To verify the model, several protocols have been developed for collecting experimental data. In a first phase, these experimental measurements were carried out in controlled environments, using loudspeakers as noise sources. In a second phase, these measurements were carried out in real environments, accounting for the specific noise sources present in each setting. The difference in sound levels between the model and reality is proven to be less than 3 dB in 75% and less than 3.5 dB in 100% of the cases examined in a controlled environment. In the real problem, in general terms and taking into account that the study is carried out on pedestrian streets, it seems that the model is able to reproduce most of the noise of anthropogenic origin.

## 1. Introduction

Noise pollution regulations mainly focus on controlling the impact produced by industrial activities, road, rail and aviation traffic. However, in recent years, cities have been impacted by a different type of noise pollution derived from commercial and leisure activities, affecting increasingly extensive pedestrian areas. Most of these activities extend into residential areas, exposing neighbours to high noise levels for much of the day and night. From a technical point of view, the problem is complex due to the variability in sound intensity levels of the noise sources, the simultaneity and directionality of the sound emission, the variability in the number of sources present and their spatial distribution, which make it difficult to apply specific control measures. From a social point of view, it is a conflict between economic, environmental and public health interests. Sometimes it is also a problem of public order. It should be added that, whatever the particular issue under study, scientific research on the subject is scarce (see, e.g., [1,2,3,4,5]).

While there is a mandatory path to follow to prevent and mitigate noise exposure under the EU Environmental Noise Directive (END) [6], in many countries, the law only specifies the acoustic quality objectives applicable to these areas, i.e., the noise levels that must never be exceeded by all the sources present. This means that public administrations are committed to carrying out periodical measurements of noise levels in the affected areas, to confirm that these limits are not exceeded or to adopt measures if they are. To achieve this, the most appropriate method is to install a permanent network of measuring instruments (sound level meters) to instantly relay information to a control and evaluation centre. This data would allow real-time noise maps to be produced using specific software [7]. In Spain, to the best of the researchers’ knowledge, such an installation only exists in the cities of Madrid and Barcelona, and they only extend over part of the affected areas [8]. The problem is that although in recent years the price of these measurement stations has been reduced considerably, this type of solution continues to be very expensive to install, maintain and operate. Very recently, other direct measurement strategies based on citizen collaboration have been launched in which participants use their personal mobile phones or low-cost sensors that they place in their homes to measure noise levels. In this context, a recent literature review about low-cost and Internet of Things (IoT) sensors in this context have been made by Picaut et al. [9]. The data collected is then sent to a web server that processes the information and generates noise maps in real time that are shared in open access. There are already some applications available that make use of this idea (see, e.g., [10]). Although interesting, this is, however, not a reliable system because it depends on the use of devices with calibrations difficult to monitor over time and on information collected by potentially affected citizens that may be impartial. In this respect, an interesting compendium and analysis of innovative solutions for noise pollution management can be found at [11].

However, an indirect approach to the problem is also possible: We can calculate a reasonable estimate of the noise level generated by determining the number of people present on the streets of these areas and their distribution. To generate this information, we only need images and a computational tool that can process them. A network of cameras suitable for this purpose is a less expensive measure than a network of sound level meters. What is more, networks of cameras already exist in many cities and densely populated areas for the purpose of surveillance and the maintenance of public order. The proposal therefore is the use of tools based on Artificial Neural Networks (ANNs) to efficiently find the crowd density in these environments from the images taken by this camera network.

Nevertheless, the propagation of acoustic waves in urban environments does not solely depend on the crowd density. It is also influenced by the geometry and planimetry of these areas. Tall buildings and narrow streets can act as acoustic canyons, making it difficult to dissipate noise and amplifying acoustic levels. The architectural features of buildings, planning and urban design therefore play a crucial role in noise management.

To address this problem, in this work, the authors propose to initiate a line of research aimed at creating applications based on numerical models in the frequency domain to reproduce the propagation of acoustic waves produced by human activity in urban areas (anthropogenic noise) in conjunction with other techniques in the field of artificial intelligence for crowd density estimation. Specifically, using the geometry of the studied area and the number of people, as well as their distribution captured by the installed cameras as input data, these tools will return as a result an estimate of the noise level at all points of the model (the noise map). In the first phase of the work, a simplified semi-analytical numerical model is proposed to reproduce the acoustic propagation produced by point sources located between two parallel reflecting boundaries, and a third one perpendicular to the previous ones, also reflecting. This model represents what is known as a Street Canyon (see, e.g., [12]), i.e., a narrow street between tall buildings. For many real situations, it can be a very representative model of the problem for receivers on a street with a medium to high density of sources emitting near ground level.

To verify this predictive model, several procedures for experimental data collection have been developed, both in controlled environments and in real scenarios using high-end commercial sound level meters and low-cost microphones to record sound pressure levels. Experimental measurements in controlled environments will be useful to calibrate the capabilities of the developed numerical model. In this case, the sources will consist of loudspeakers emitting noise of known intensity characteristics. To take measurements in real scenarios, two streets were selected in the city of Las Palmas de Gran Canaria (Canary Islands, Spain), where the geometry can be approximated to a Street Canyon and where the concentration of people is high and variable throughout the week. For this purpose, outdoor kits were manufactured that can be anchored to the façades and collect data over long periods of time. These kits consist of a microphone (connected to a sound level meter inside), an RGB-D camera and an anemometer. Self-developed software was used to process the recorded results (sound and image).

In the literature, contributions in line with this proposal are scarce. In relation to the problem that arises and also proposing indirect strategies to obtain estimates of noise levels, the work of Ballesteros et al. [1] should be quoted. The authors present regression models for estimating the noise level produced by leisure activities in some streets of Madrid and Cuenca (Spain), using the number and type of businesses as variables. Likewise, the work of Genaro et al. [13] is very relevant as it studies the use of ANNs to model the noise in twelve streets of the city of Granada (Spain), produced mainly by road traffic. More recently, another interesting contribution in line with the study of crowd density and its influence on the noise level in urban areas is that of Meng and Kang [14], who study the influence of crowd density on the noise level in pedestrian streets where commercial activities take place, by measuring noise levels and taking photographs on streets in the city of Harbin (China). To determine the number of people per square metre, they used the photographs taken and a questionnaire seeking to study the frequency of visits to the commercial area that was given to pedestrians whilst measurements were being taken. They use a technique based on dividing the study area into grids to position pedestrians with respect to the measuring device. Along the same lines and which may also be of interest, there is another contribution by Meng et al. [15] in which the effect of street markets on noise levels and acoustic perception is studied based on crowd density and street market zoning. Again, to determine the number of people per square metre, they use photographs. Finally, a fairly novel application is that of Elvas et al. [16]. In this publication, night-time urban noise patterns in the city of Lisboa (Portugal) are analysed, and different areas are identified using GPS data from mobile phones.

However, the tools proposed to be developed in this line of work have a double objective: (1) to permit the evaluation of the acoustic levels based only on the visual information recorded by the cameras and whether these levels exceed the quality limits established by law or not, and (2) more importantly, to be used as simulation software that allows the incorporation and evaluation of some corrective measures.

The structure of this paper is as follows: Section 2 provides a description of the mathematical problem posed, as well as the hypotheses considered. It also describes the mathematical basis of the model developed, the expression that solves the problem posed, the convergence accelerator that allows results to be obtained in an acceptable time, the mathematical modelling of the human voice spectrum and its intensity level adjustment. The Artificial Neural Network used to obtain crowd density from images is described in Section 3. Finally, the two experiments carried out for the verification of the developed model are described in detail in Section 4, which also describes the processing of the data collected during the experiments, their comparison with the values provided by the model and the results obtained.

## 2. Description of the Mathematical Model

### 2.1. Model Assumptions

**Figure 1:**
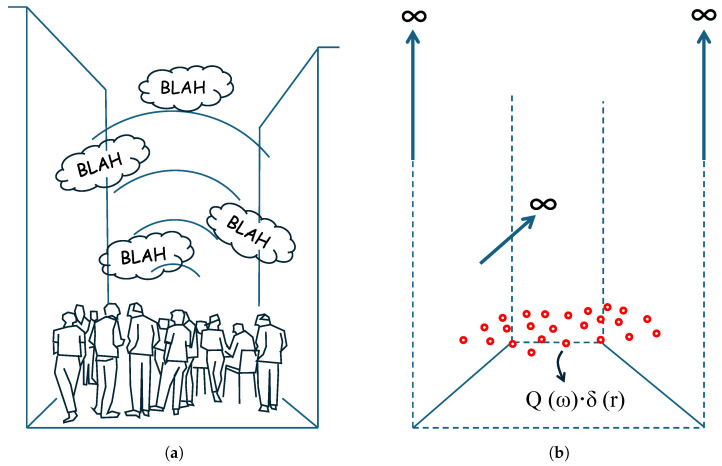
(**a**) Representation of the real scenario posed in the problem. (**b**) Conceptual model proposed for analysis.

The problem that arises is to determine the sound pressure at a series of points (receivers) generated, in a real case, by people emitting sounds in a pedestrian street bounded by two vertical and parallel façades. In the mathematical model, people are represented by point sources emitting sounds with a spectrum similar to that of human speech in a domain bounded by three reflective boundaries (Street Canyon). The street has infinite height and length, and is of known width. Figure 1 shows a representation of the real scenario posed in the problem.

The assumptions considered in the proposed mathematical model are listed below:

1. All surfaces, both façades and floors, are considered to be perfectly reflective surfaces.
2. Noise sources are considered omnidirectional. The Dirac delta function $\left(\delta \right)$ is used to model them mathematically. $Q\left(\omega \right)$ represents the intensity of the source; it is a function of frequency and shall be calibrated from a reference spectrum to be established for this purpose.
3. The sound propagation medium (air) shall be treated as a perfectly elastic and compressible continuous medium with negligible viscosity and isotropic behaviour.
4. The problem of acoustic wave propagation in environments not far from the noise source is studied. Thus, the propagation medium can be considered homogeneous.
5. The effects of wind on sound propagation are neglected (air at rest).
6. The disturbances produced by the sound propagation are small enough for the changes in pressure and density to be minimal compared with the values at rest.

The physical and mathematical formulation of the problem is approached from harmonic elastodynamics. As will be seen, in order to model a spectrum equivalent to that of a person talking, the spectrum of a theoretical A-weighted pink noise is used, to which the intensity level is adjusted as a function of the crowd density by means of a regression curve.

### 2.2. Basic Equations

The description of the mathematical model will begin by posing the wave equation in the frequency domain, which describes the propagation of acoustic waves in three dimensions. The wave equation can be stated in terms of any of the acoustic variables: pressure, density and displacement or velocity. For this work, pressure is taken as the dependent variable. Thus, the equation governing the propagation of harmonic acoustic waves is (Helmholtz equation).

(1) $$\nabla^{2}p+k^{2}\cdot p+\delta \left(\vec{r}\right)=0$$

where

- ∇ is the divergence operator in 3D problems: $\nabla \equiv \frac{\partial }{\partial x}\vec{i}+\frac{\partial }{\partial y}\vec{j}+\frac{\partial }{\partial z}\vec{k}$;
- *p* is the sound pressure at any point;
- The constant k=ω/c is defined as the wave number. Where, in turn, ω=2πf is the angular frequency, *f* is the frequency in Hz and *c* represents the wave propagation speed. It will depend on the thermodynamic characteristics of the medium (temperature, pressure and density) and define the elastic properties of the fluid. For a temperature of 20 °C, *c* will have a value of 343.5 m/s [17];
- The Dirac delta function $\delta \left(r\right)$ is related to the presence of internal harmonic point pressure sources with a time variation of the type of $e^{-i\omega t}$.

To integrate and obtain a solution to the governing Equation (1), it is necessary to impose boundary conditions. In the proposed model, façades and ground are considered perfectly reflecting, which can be described mathematically in terms of the pressure flux on those boundaries as follows:

(2) $$\frac{\partial p}{\partial n}=0$$

The solution must verify the equation governing the propagation of acoustic waves in a homogeneous, non-viscous and linear medium in the frequency domain (1) and also fulfil the conditions imposed on the boundaries of the domain under study (2).

**Figure 2:**
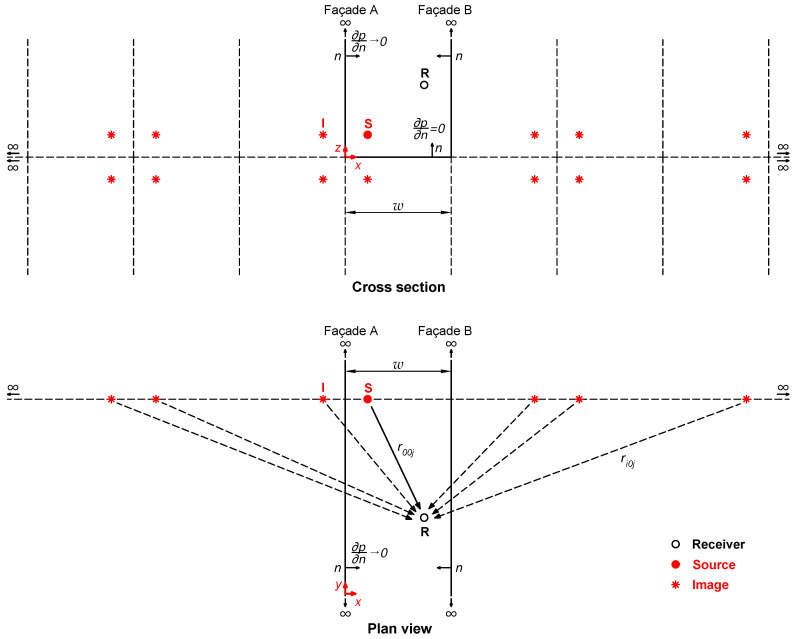
Graphic representation of the solution for the sound propagation in a Street Canyon with perfectly reflecting boundaries.

Back to the problem at hand, the sound pressure at any point $\left(x,y,z\right)$ in a Street Canyon model caused by a source at a point $\left(x_{s},y_{s},z_{s}\right)$ inside the model (Green Function) can be calculated using the image source method (Figure 2).

The succession of image sources becomes infinite, but it allows us to obtain a solution to the Street Canyon model by which the sound pressure at any point between the façades can be written as follows (see, e.g., [18]):

(3) $$p^{sc}\left(k,x,y,z\right)=\frac{1}{4\pi }\cdot \sum_{j=0}^{1}\left[\frac{e^{-ikr_{00j}}}{r_{00j}}+\sum_{n=0}^{N_{w}}\left(\sum_{i=1}^{4}\frac{e^{-ikr_{i0j}}}{r_{i0j}}\right)\right]\;\left(N_{w}\to \infty \right)$$

being:

(4) $$r_{i0j}={\left[x_{i}^{2}+y_{0}^{2}+z_{j}^{2}\right]}^{1/2}\;\left(i=0,\ldots ,4\;;\;j=0,1\right)$$

obtaining $x_{i}$, $y_{0}$ and $z_{j}$ from:

(5) $$\begin{array}{ccc} x_{0}=x-x_{s} & \;y_{0}=y-y_{s}\; & z_{0}=z-z_{s}\\ x_{1}=x+x_{s}+2nw & \; & z_{1}=z+z_{s}\\ x_{2}=x+x_{s}-2\left(n+1\right)w\\ x_{3}=x-x_{s}-2\left(n+1\right)w\\ x_{4}=x-x_{s}+2\left(n+1\right)w \end{array}$$

where *w* is the width of the Street Canyon and $N_{w}$ the number of virtual image sources in the horizontal direction placed in such a way that the zero pressure flux (2) on both façades and on the street ground can be verified simultaneously.

Other more straightforward solutions that can be particularised from Equation (3) and that will be employed in other sections of the paper are as follows:

- Solution for a free-field model, where no reflective surfaces are present:

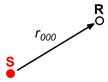
        $$p^{ff}\left(k,x,y,z\right)=\frac{1}{4\pi }\cdot \frac{e^{-ikr_{000}}}{r_{000}}$$        (6)

- Solution for a half-space model, where one reflective surface is present:

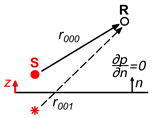
      

$$p^{hs}\left(k,x,y,z\right)=\frac{1}{4\pi }\cdot \sum_{j=0}^{1}\frac{e^{-ikr_{00j}}}{r_{00j}}$$

       (7)

- Solution for an open canyon model, where two reflective surfaces are present (floor and one façade):

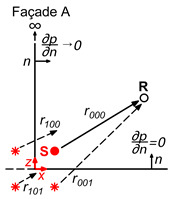
 

$$p^{oc}\left(k,x,y,z\right)=\frac{1}{4\pi }\cdot \sum_{j=0}^{1}\left(\frac{e^{-ikr_{00j}}}{r_{00j}}+\frac{e^{-ikr_{10j}}}{r_{10j}}\right)\;\;\;\left(n=0\right)$$

   (8)

### 2.3. Convergence Accelerator

The generated series for the Street Canyon solution has a very slow (sublinear) convergence speed, so accelerator algorithms are used to reduce the computation time of $p^{sc}$ to a manageable time. The Aitken–Shanks $\Delta^{2}$ algorithm is applied [19,20]. This procedure makes it possible to transform the original series into an equivalent series with a higher convergence speed. For the problem under consideration, when compared with other series acceleration techniques (see, e.g., [21]), this transformation was chosen because it leads to a very simple algorithm with an acceptable precision and computational cost. As is well known, the Aitken–Shanks transformation can be applied successively and an almost linear convergence can be achieved.

**Figure 3:**
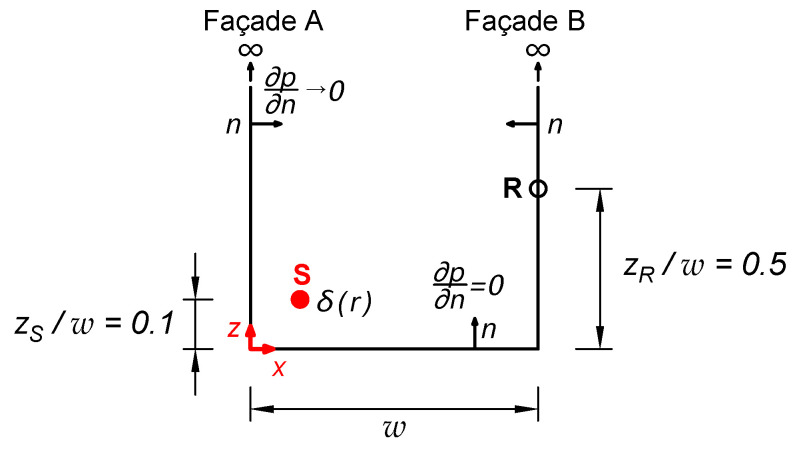
Representation of the problem posed for the study of the Aitken–Shanks method.

**Figure 4:**
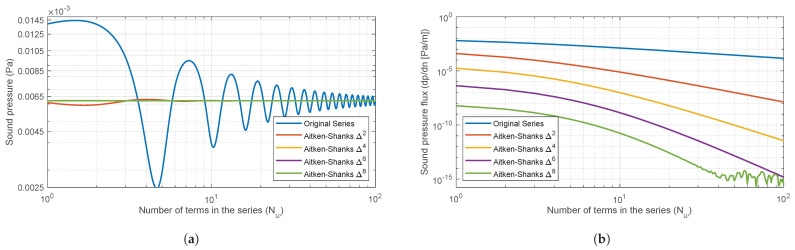
Green Function and Aitken−Shanks convergence for Street Canyon problem (Figure 3). (**a**) Sound pressure at the façade B and (**b**) Normal derivative of the sound pressure at the façade B, plotted against the number of terms in the series for a value of $\frac{w}{\lambda }=2.9$. A logarithmic scale was employed on abscissa axes.

**Figure 5:**
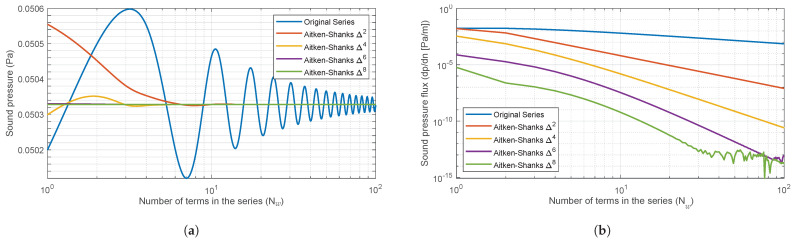
Green Function and Aitken−Shanks convergence for Street Canyon problem (Figure 3). (**a**) Sound pressure at the façade B and (**b**) Normal derivative of the sound pressure at the façade B, plotted against the number of terms in the series for a value of $\frac{w}{\lambda }=15$. A logarithmic scale was employed on abscissa axes.

To determine the order of the transform and the number of terms $\left(N_{w}\right)$ of the optimal series, an exhaustive study of the Aitken–Shanks method $\Delta^{2}$, $\Delta^{4}$, $\Delta^{6}$ and $\Delta^{8}$ was carried out for a wide range of ^*w*^$\;{/}_{\lambda }$ values (λ is the wavelength) and relative positions of source and receiver, as can be seen in Figure 3. To visualise the behaviour of these transforms, some of the results for two values of ^*w*^$\;{/}_{\lambda }$ are shown in terms of the sound pressure and its Normal derivative at the façade contour (Figure 4 and Figure 5), which must have a value of zero.

It can be observed that as the frequency increases, the transforms become increasingly unstable with a decreasing number of terms in the series. In order to prevent the convergence of the model from becoming unstable, 50 terms of the Aitken–Shanks $\Delta^{4}$ series transformation will be used. This approach ensures that the model converges in a perfectly admissible time frame and that the resulting errors are consistently smaller than $10^{-7}$.

### 2.4. Calibration of Model Point Sources and Procedure to Obtain Sound Pressure Level

**Figure 6:**
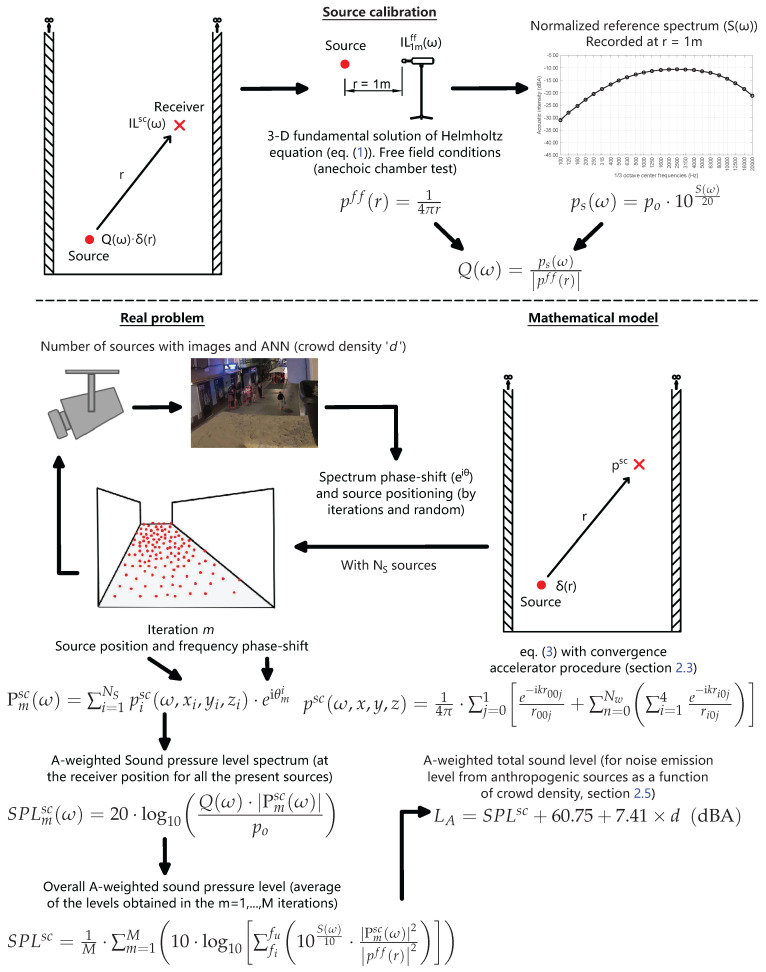
Calibration of model point sources and procedure to obtain the sound level at the receiver’s positions.

This section describes the mathematical procedure used to calibrate the noise spectrum of the sources within the model and to obtain the sound level at any point from that reference spectrum. To enhance comprehension of the procedure, Figure 6 graphically describes the calibration process and the calculation of the final sound level.

In the figure, $S\left(\omega \right)$ represents the reference spectrum employed, recorded in decibels (dBA), at a distance of 1 m from the source and with free-field conditions. It is necessary to obtain it in terms of sound pressure $p_{s}\left(\omega \right)$, with the reference pressure for this conversion being the human hearing threshold ($p_{o}=20\;\mu$Pa). The calibration factor, denoted here by $p^{ff}$ and calculated under free-field conditions, Equation (6) for the general case (fundamental solution), can obviously be calculated with other boundary conditions that will depend on the experimental constraints under which the reference spectrum available for the problem to be simulated can be recorded.

For a given degree of crowd density (the number of point sources), the sound pressure level at the observation point (the receiver) is obtained by combining the sound pressure values, calculated for each source from this calibration and the corresponding Green Function $\left(p^{sc}\right)$. The value obtained is subjected to slight variation depending on the precise position of each source in relation to the receiver and the phase shift between the spectra emitted by each, with these effects being more pronounced for a smaller number of sources. It is important to note that the number of sources is calculated from crowd density data obtained from images captured at regular intervals. Given that the exact position of each source in a given area is uncertain and changes with time, the determination of the noise level using this model is performed via the calculation of the mean value from a Monte Carlo simulation. The simulation involves random positioning of the sources within the occupied area and incorporates random phase shifting between the noise spectra of each source. The sound spectrum level recorded at the observation point for the calculation iteration *m*, in terms of spectral density and in dBA, is designated as $SPL_{m}^{sc}\left(\omega \right)$. The normalised average equivalent sound pressure level is denoted by $SPL^{sc}$, and it represents the average of the combinations of sound pressure levels $SPL_{m}^{sc}\left(\omega \right)$ obtained for each frequency and for each iteration in the usual way. The initial and final frequencies of the frequency range, designated by $f_{i}$ and $f_{u}$, respectively, are also employed in this context.

### 2.5. Definition of Anthropogenic Noise Spectrum

A significant source of uncertainty in attempting to solve the proposed problem is the identification of the emission spectrum exhibited by individuals during conversation or leisure activities. One of the most frequently utilised standards is the ANSI S3.5-1997 [22] that provides a series of standardised spectra that simulate the sound emitted by people during a conversation with four levels of acoustic intensity: “Normal”, “Raised”, “Loud” and “Shout”.

**Figure 7:**
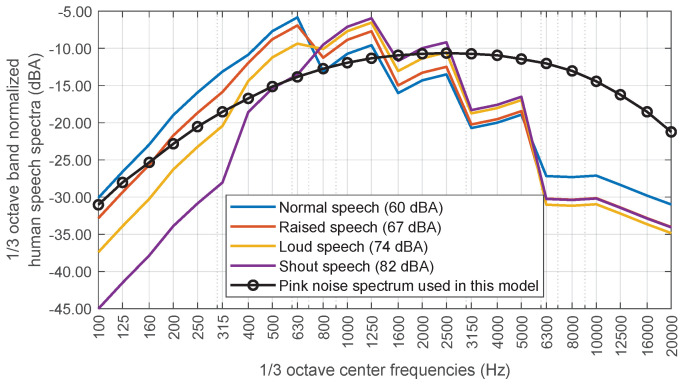
Comparative graph between the human voice spectrum used in the model and the spectra provided by the ANSI S3.5−1997 standard for 4 sound intensity levels, all of them normalised to 0 dBA.

However, for the sake of simplicity, a theoretical A-weighted pink noise spectrum is used to represent the frequency content of the human voice. The original ANSI spectra are given in SPL [dB] for octave frequencies. Thus, in order to compare the proposed spectrum to the ANSI spectra, the latter must be interpolated at one-third octave frequencies and then the A-weighting applied. In Figure 7, the A-weighted spectrum used and the modified ANSI spectra are shown, where all of them have been normalised to 0 dBA. It is shown that they are reasonably similar.

The key question at this point, and one of the major uncertainties of the proposed model, is to quantify the sound intensity level of the sources in order to reproduce a real anthropogenic noise problem. As already advanced in the previous section, the source sound intensity level was established in this study as usual: a single source emitting in a free field and measured at 1 m. In the literature, the existing information about human sound sources does not go beyond the spectra and sound levels provided by the usual application standards for different discrete speech levels of a single human voice: Normal (60 dBA), Raised (67 dBA), Loud (74 dBA) and Shout (82 dBA). When people are involved in a social activity, the speech level of each person is difficult to establish. Each person decides to speak at a given speech level depending on the context. Somehow, each person adapts the speak level to the environment in order to convey an intelligible message by considering aspects such as the number of listeners (which are silent), distance to listeners, background type and level of noise, the type of social activity (leisure, academic, work, etcetera), age, culture, language and so on. The number of factors affecting the speech level is large, difficult to establish and difficult to quantify, mainly because, in the end, it is a social activity. In this work, the authors are only concerned with leisure activities on pedestrian Street Canyons, where people are relatively homogenous as sound sources and two factors affecting the sources are relatively easy to measure: the surface area and the number of people in this surface area.

Taking all of this into account, the following working hypothesis is proposed: the sound intensity level of a human source is a function of the density of the people in the street. It is reasonable to think that people, to make their conversation intelligible, raise the level of their speech some decibels above the background sound level, which can be related to the density of people as long as all the people are involved in the same activity.

**Figure 8:**
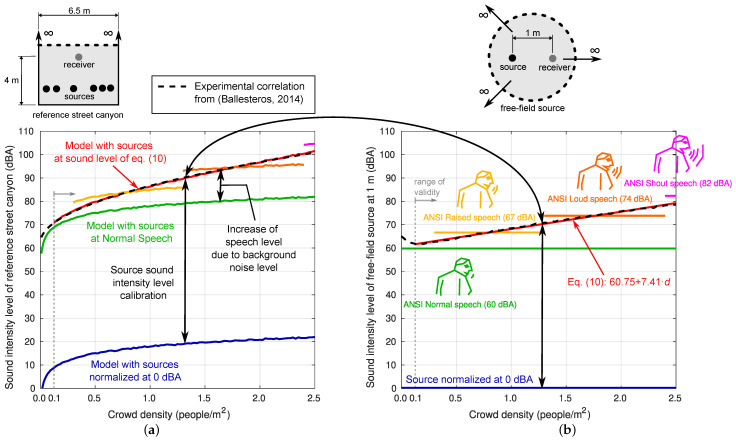
Adjustment of the intensity value of each source from Ballesteros et al. [1]. (**a**) Results concerning the reference Street Canyon of 6.5 m width where different relationships between the sound intensity level (dBA) measured at 4 m height produced by a variable density of people per square metre are shown. (**b**) Different theoretical sound intensity levels of an A-weighted pink noise sound source in free-field conditions at 1 m.

In this work, a simple fitting procedure is proposed by solving the inverse problem using experimental information available in a few published papers. In this sense, some results presented by Ballesteros et al. [1] are very well adapted to this task. These authors present a regression equation on experimental data that allows obtaining the sound level from the crowd density in a wide range of values of this variable and in a street that can be well adapted to the Street Canyon model proposed. This regression equation is as follows (Figure 8a):

(9) $$L_{Aeq}=62.15+24.49\times \sqrt{d}\;\;\;\;\;\;\;\left(\mathrm{dBA}\right)$$

where $L_{Aeq}$ is the continuous equivalent sound level in dBA and *d* is the density of people per square metre. These authors clarify that this regression curve was obtained from data taken experimentally in a specific street in the city of Cuenca, Spain (Dr. Galindez Street) with a significant number of leisure places. The dimensions of this street (*w* = 6.5 m) and the height of the receiver for which the measurement is taken (*h* = 4.0 m) are known.

The procedure is explained graphically in the Figure 8. On the left-hand side of the figure, results concerning the reference Street Canyon of 6.5 m width are shown. Different relationships between the sound intensity level (dBA) measured at 4 m height produced by a variable density of people per square metre are shown. For now, note only that the black dashed line corresponds to the reference correlation experimentally obtained in Ballesteros et al. [1]. This empirical correlation contains a statistical measure of all the factors described above regarding the human sound sources behaviour, and also the sound propagation phenomena within the urban canyon (the street width and receiver and sources position).

On the right-hand side of the figure, different theoretical sound intensity levels of an A-weighted pink noise sound source in free-field conditions at 1 m, $L_{As}^{ff}\left(1m\right)$, are shown. The blue line shows a source normalised at 0 dBA, with no dependency of the crowd density. The blue line on the left-hand side of the figure shows results with the reference Street Canyon model if such a source is used. For these results, at each crowd density level, the average of 30 iterations performed by randomly distributing the sources in the occupied zone and including a random phase-shifted value in their emission spectra is taken. The observed dependency between the sound intensity level and the crowd density is only due to the number and position of sources. An increase in the sound intensity level of each source in, e.g., 10 dBA, implies an increase in the same value of the sound intensity level measured at the receiver for all crowd densities. An increase of 60 dBA of the sound intensity level of each source leads to the green lines shown on the right-hand and left-hand sides of the figure. In this case, the sound intensity level of each source is similar to the ANSI Normal speech. It is observed that the prediction of the model if all sources are emitting at “ANSI normal speech” for all crowd densities greatly differs from the experimental results. Experimental results can be explained by the model only if the sound intensity level of each source linearly increases as the crowd density increases. By fitting the difference between Ballesteros et al.’s [1] correlation (the black dashed lines) and the model prediction with 0 dBA sources (the blue solid line), the following linear regression with a coefficient of determination $\left(R^{2}=0.9966\right)$ is obtained:

(10) $$L_{As}^{ff}\left(1\;m\right)=60.75+7.41\times d\;\;\;\;\;\;\;\left(\mathrm{dBA}\right)$$

which is valid for d≥0.1 people/m^2^. This type of source (the red solid line) now responds to the crowd density (the background noise) by linearly increasing the sound intensity level, and the experimental results are accurately predicted by the model. It is an interesting, simple, consistent and practical conclusion that can generally be applied to characterise the behaviour of anthropogenic sources in problems of this type.

In any case, before concluding, it is important to point out that, although in this model all sources are omnidirectional, in reality this is not entirely true in the problem we are studying. For “normal” speech levels and frequencies below 1 kHz, a person can be regarded as a practically omnidirectional source in all three dimensions [23,24]. At overall sound pressure levels, the vertical plane (2D) does exhibit some degree of directionality, though the differences are less than 10 dBA [25]. In the horizontal plane, however, there is minimal directionality. At higher levels (“loud speech”), there appears to be a slight increase in the directionality of the sound, as evidenced by the findings of [23,25].

### 2.6. Effect of Trapped Modes of Street Canyon Model in the Intensity Noise Level Prediction

**Figure 9:**
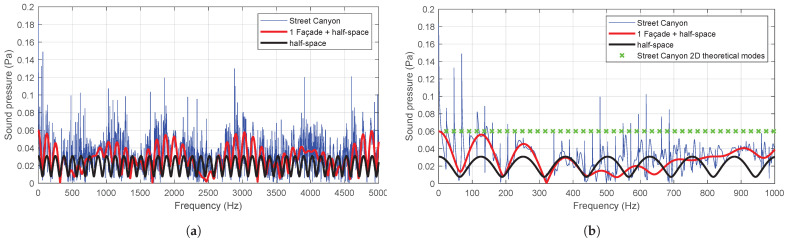
Trapped modes of Street Canyon model. (**a**) Representation of the model response using spectral density for a single source with different number of reflecting surfaces and (**b**) zoom 0–1000 Hz for the representation of the theoretical horizontal modes of a Street Canyon.

In this problem, the classical effect of the existence of natural frequencies and eigenmodes is observed as a consequence of the presence of reflective surfaces. These discrete modes represent an acoustic resonance and are often called “trapped modes” [26], and it is therefore necessary to analyse the extent to which these modes can alter the calculation of the overall sound pressure level, bearing in mind that it is usual to calculate it on the basis of one-third octave bands spectra. In this way, the behaviour in each band can be characterised through its characteristic frequency. However, this approach inevitably entails a loss of information. Therefore, the aforementioned spectrum will be employed as the emission spectrum of the sources in a spectral density analysis with the numerical model, using a bandwidth of 1 Hz. Furthermore, this procedure allows for the acquisition of the spectrum in one-third octave bands, and to use their centre frequencies as characteristics of the model response. This enables the full range of information and responses to be captured in each vibration mode. The occurrence of these natural frequencies is illustrated in Figure 9. It should be noted that Figure 9b, which has been magnified for clarity, illustrates the horizontal theoretical modes of a Street Canyon ($f_{n}=$^*n*·*c*^/_2·*w*_).

**Figure 10:**
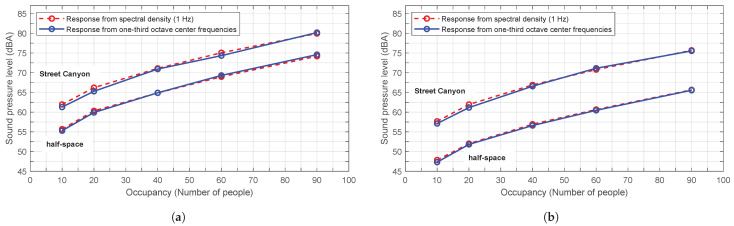
Comparative plot between the sound pressure level calculated from the spectral density and from one-third octave band center frequencies, for different number of sources in the described problem. The average result of 10 calculation iterations is shown. (**a**) Receiver 1. (**b**) Receiver 2.

However, the presence of multiple sources, the randomness of their arrangement, the use of phase-shifted frequencies between them and the evaluation over a high number of computational iterations result in very similar average responses. Figure 10 illustrates the response of the numerical model to the simulation of a street of 7.5 m width, with two receivers placed in different positions at a height of 5 m and 15 m, respectively. The sound pressure level is obtained for 10, 20, 40, 60 and 90 people distributed in a 3.5×20 m^2^ area, using spectral density and one-third octave bands for both the Street Canyon model and the half-space model. The results presented here are the average of 10 computational iterations, each with a random arrangement and phase shift.

It can be inferred that the specific treatment of this problem, in averages, does not significantly differ whether it is conducted in one-third octave bands or from the spectral density, and trapped modes are of little relevance.

## 3. Crowd Density Estimation from Computer Vision

An ANN capable of detecting people in images [27] is employed to obtain the crowd density. The system is fed with images captured by a network of cameras that periodically photograph the areas under study.

The ANN used in this work, the Ultralytics YOLOv8 model [28], is based in the original single stage detector architectures described by Joseph Redmon and Ali Farhadi at the University of Washington [29,30,31]. It is a real-time image segmentation and object detection model based on deep learning and computer vision. Among the object detection architectures compared, it demonstrates a high level of performance and exhibits considerable versatility with respect to the hardware platform and operating system employed. We adopted the pre-trained medium model for the detection of people in images.

About the general performance of the YOLOv8 object detection model, in the case of the class “person”, the mean average precision from Intersection over Union (IoU) thresholds of 0.5 to 0.95 (mAP50-95) on the Ultralytics COCO validation dataset [32] exceeds 70% [33]. The IoU parameter is a metric used to quantify the accuracy with which a predicted boundary, such as a bounding box in object detection, matches the real boundary of an object. In essence, the IoU measures the degree of overlap between the predicted and true areas, providing a simple but effective metric for evaluating the performance of localisation algorithms [34].

**Figure 11:**
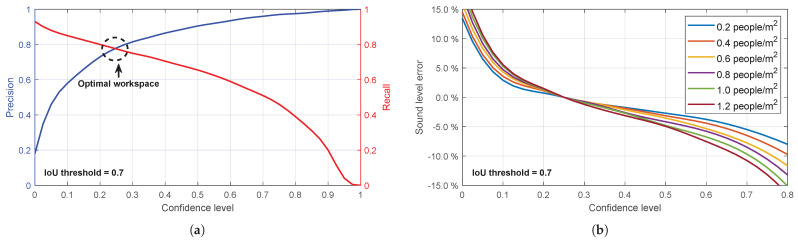
Analysis of the performance of YOLOv8 in the detection of the object class “person”. (**a**) Precision−recall curves for different confidence levels extracted from the evaluation of YOLOv8 with COCO dataset [32]. (**b**) Influence of possible errors in the detection of people on the response of the proposed acoustic model as a function of confidence level, for different crowd densities.

In low-light conditions, the performance of the YOLO architecture, even without adaptation, maintains mAP values close to 70% (see, e.g., [35]). These metrics, while informative, are obtained for varying confidence thresholds. However, for practical use as intended, it is necessary to determine the most appropriate value for this parameter. For this purpose, precision–recall curves for different confidence levels are extracted from YOLO (Figure 11a).

It is easy to see that the confidence level value that reports people counting closest to the real value should be between 0.2 and 0.3. In this interval, False Negatives and False Positives in the verification process eventually compensate each other. However, in this work, a series of tests are performed in each scenario, and the conclusion is that a confidence level of about 0.2, although apparently somewhat low, is the best to adjust the response of this ANN. The number of images analysed for this task exceeds three hundred, randomly selected from the images captured during the measurement campaign on Sargento Llagas Street, in which the images were captured with a one-minute time step. Throughout this process, the default value of IoU = 0.7 is adopted.

At this point, it is also interesting to analyse the influence that eventually errors in the detection of people have on the response of the proposed acoustic model. Figure 11b aims to show this sensitivity as a function of the confidence threshold used by the detection tool. It represents the error made in the sound intensity level reported by the numerical acoustic model for different values of the crowd density predicted by YOLOv8 and the one that eventually exists in reality depending on this confidence threshold. The difference between both values will be higher for confidence levels outside the mentioned interval (0.2–0.3), but always within a margin of less than 15% even in the most extreme assumptions. This is an interesting fact that allows us to calibrate the utility margins of the procedure for this application.

**Figure 12:**
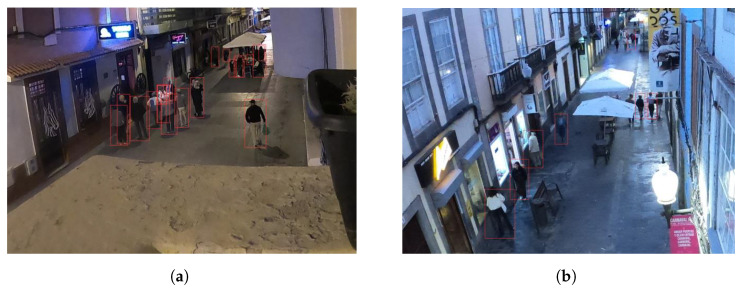
Neural network output for crowd density estimation. Images related to the validation procedure to be presented in Section 4.2. (**a**) Sargento Llagas Street. (**b**) Cano Street. Both located in Las Palmas de Gran Canaria, Spain.

Figure 12 illustrates an example of the manner in which the network presents the outcomes of the people detection process. In the processed images, the labels indicating the results of the detection process (the classification of the detected object and confidence level) were deactivated. In addition to the images displaying the detected persons, the network returns a text file for each processed image, wherein all the information necessary to calculate the parameters characterizing the crowd is displayed: the number of people, position in image coordinates (the bounding box) and density according to each occupied area.

In line with the proposal made in this paper and also using YOLO, at this point, it is worth mentioning the work published by Fredianelli et al. [36], which orients this tool to the recognition and counting of vehicles according to the requirements of the CNOSSOS-EU noise assessment model. Regarding the previous experience of the group in the use of this software, the YOLO people detector (specifically YOLOv8) has already been used by some of the authors of this paper integrated with the ByteTrack people tracker [37] to detect individuals in challenging scenarios, such as trail races, in re-identification tasks [38,39]. In these scenarios, people detection is particularly challenging due to cluttered backgrounds and difficult lighting conditions, including night-time illumination.

## 4. Experimental Validation

This section outlines the validation experiments conducted on the proposed model. They were carried out at two levels: (1) a validation experiment with artificial noise sources, wherein the number, position, sound level and noise spectra of the sources are known, and (2) a validation experiment in a real environment with noise of anthropogenic origin. The primary objective of the first experiment is to ascertain the model’s capacity to reproduce acoustic propagation in a real Street Canyon. The second experiment assesses the comprehensive proposed model and its analytical assumptions.

### 4.1. Validation with Controlled Artificial Noise Sources

The experiment was conducted in a passageway within the Edificio de Ciencias Básicas of the Tafira Campus of the University of Las Palmas de Gran Canaria. The passageway is formed by the façades of two three-storey buildings and is 8.30 m width. The configuration of sources and receivers is described in the experimental setup (Section 4.1.3).

#### 4.1.1. Devices and Materials

Artificial noise sources: Four loudspeakers of model WonderBoom-2 from the manufacturer Ultimate Ears (Irvine, CA, USA) are utilised. The speakers were modified, the Bluetooth connection was cancelled and the speakers were wired in order to facilitate direct connection to an amplifier and enhance control over the speaker and the signal it emits. Four height-adjustable stands are used to support the speakers.

Sound system: The signal sent to the loudspeakers is pink noise. It is reproduced with a sound system model XC-IS21T and an amplifier model M-IS21 of the manufacturer Pioneer (Tokyo, Japan), to which the loudspeakers are directly connected.

Receivers: Four Beyerdynamic (Heilbronn, Germany) model MM1 microphones with corresponding windscreen and stand, supplied by the manufacturer. All have a sensitivity of −36.5 dB at 1 kHz equivalent to 15 mV/Pa (measured at 1 m). Four sound level meters from the manufacturer Hottinger Brüel & Kjaer (Nærum, Denmark) are also used. Two sound level meters of the model 2250 (G4), one model 2270 (G4) and one model 2250 Light. Two of the sound level meters are equipped with the weatherproof microphone kit model UA-1404, weatherproof microphones model 4952 and 3 m extension cable model AO-0697-D-030. The other two are equipped with the outdoor kit, one with microphone model 4189 and the other with microphone model 4950, both with preamplifier model ZC-0032 and 90 mm windscreen model UA-1650. All sound level meters have a sampling rate of 48 kHz [40].

Sound interface: A Roland (Osaka, Japan) model Octa-Capture audio interface is used. This device captures the analogue signal and converts it to a digital signal with a range between −1 and 1, equivalent to −0.775 V and 0.775 V. The device’s sensitivity is set at 50 dB and its sampling rate is 44,100 Hz. The interface is connected to a laptop computer via a USB connection.

**Figure 13:**
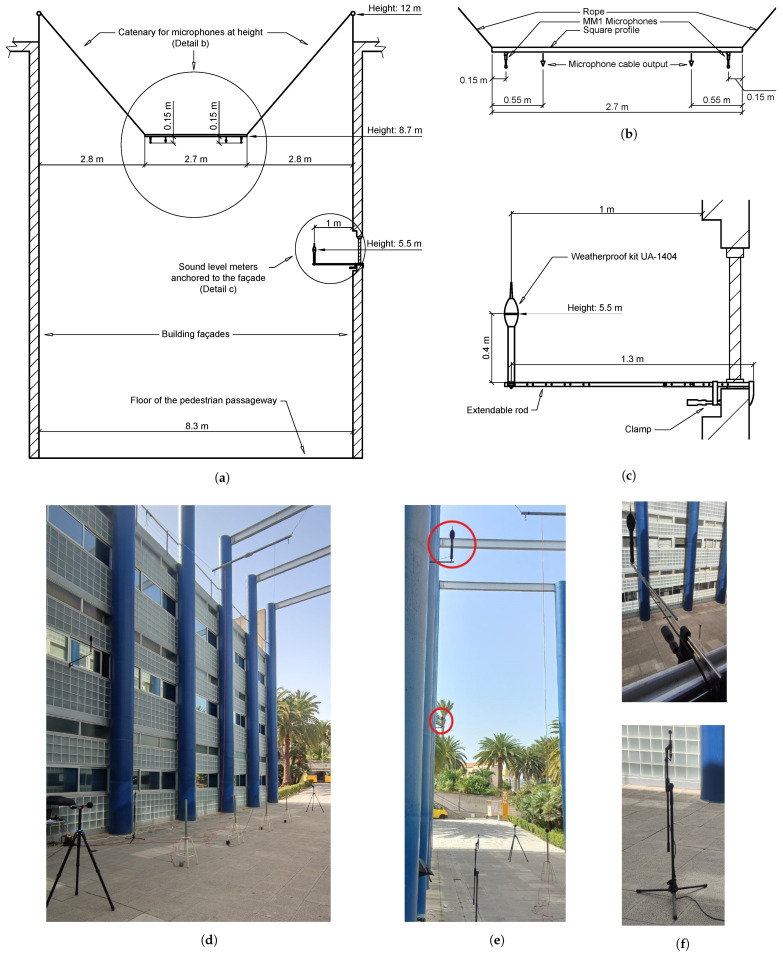
Setup of the experimental validation. (**a**) General view of the mounting of receiver brackets at height. (**b**) Detail of the microphone stand in height. (**c**) Detail of the brackets for sound level meters anchored to the façade. (**d**) Image of the experimental setup. (**e**) Image of the location of the two sound level meters anchored to the façade. (**f**) Images of the brackets for receivers.

Mounting (see Figure 13): Two tripods of model UA-0801 are employed for the installation of two sound level meters. The remaining two meters are anchored to the façades with a self-made fastening system (Figure 13c,f). A tripod is employed as a stand for one of the microphones. The fastening system for the two microphones located at height consists of a square metal profile anchored at height by means of a rope, in the shape of a catenary (Figure 13a,b).

Recording equipment: A laptop computer with the sound interface controller (Octa-Capture Control Panel) and the self-developed software installed, which records the signal from the microphones and processes it to obtain all sound levels presented. It should be noted that since this is a direct validation of the physical phenomenon, in this experiment, it is considered convenient to treat all acoustic levels for “zero” frequency weighting, or Z-weighting (i.e., no weighting across the audio spectrum).

Just before the start of the experiment, the sound level meters were calibrated utilising a sound level meter calibrator, model 4231. The correct operation of the microphones was validated with the aforementioned sound level meters in a controlled laboratory test.

#### 4.1.2. Emission Spectrum and Directionality of Loudspeakers

Prior to undertaking the validation experiment, it is essential to ascertain the real characteristics of the noise spectrum of the sources employed: their sound level, frequency composition and directionality.

For this purpose, a preliminary test is conducted in an open field (as the research group currently lacks anechoic chambers) so that the reference solution for the numerical model will be the Green Function corresponding to the half-space, Equation (7). The test is carried out in a large field devoid of reflective surfaces, with the exception of the ground, which is flat and uniform across a vast area from where the set is established. A 1.4 m high base that rotates on its axis is used as a support for the loudspeakers. The rotating base is placed on a goniometer.

Four microphones are utilised for the purpose of recording the spectrum emitted by the loudspeakers, with the corresponding windscreen and stand also employed. The microphones are positioned at 0°, 90°, 180°and 270° rotation angles relative to the loudspeaker. All microphones are positioned at a height of 1.4 m and at the reference distance from the loudspeaker (1 m).

**Figure 14:**
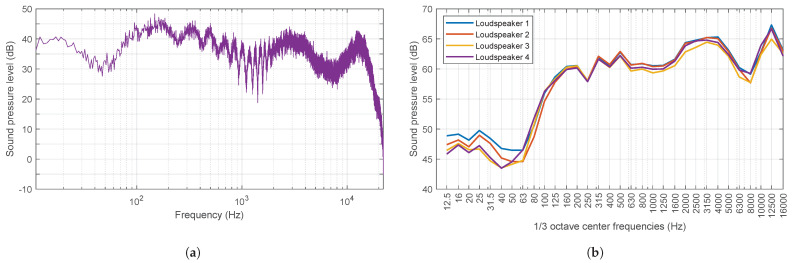
Recorded spectra. (**a**) Equivalent continuous density spectrum taken over 15 s period for loudspeaker 4 in 60° direction. (**b**) Equivalent continuous spectra recorded for the 4 loudspeakers in one−third octave bands. Both represented as $L_{Zeq}$ (dB). A logarithmic scale was employed for the abscissa axis.

The signal transmitted to the loudspeakers is pre-recorded pink noise. Records are taken at 30° intervals until the loudspeaker has completed one full rotation, repeating the procedure for the remaining loudspeakers one at a time. Figure 14a illustrates, as an example, the equivalent continuous density spectrum recorded for one of the loudspeakers in one of the directions, utilising a bandwidth of 1 Hz and over the 15 s of measurement. Figure 14b illustrates the spectra recorded for the four loudspeakers, over the 15 s measurement period and for all directions, in one-third octave bands.

It can be observed in the recorded spectrum that, despite the signal transmitted to the loudspeakers being pink noise, the recorded sound is not. This discrepancy can be attributed to the filtering effects of the device’s hardware and the environment (half-space) on the transmitted signal. From the perspective of the experiment’s intended purpose, this is not a drawback. This experimental spectrum will be fed to the model in order to calibrate the sources from the Green Function for the half-space, Equation (7). From this point on, the essential part of the procedure followed to calculate sound levels is that described in Section 2.4.

**Figure 15:**
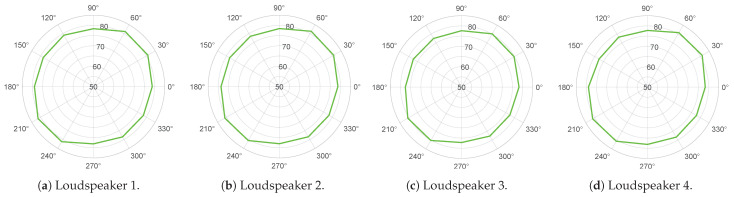
Directionality plots of the four loudspeakers WonderBoom-2. Represented as $L_{Zeq}$ (dB).

On the other hand, the directionality plots of the loudspeakers are shown in Figure 15. It can be concluded that the four loudspeakers exhibit a highly similar response in all directions within the two-dimensional plane, both in terms of spectra and overall intensity levels. It is evident that a slight deformation in the circumferences is consistently observed in the same direction for the four loudspeakers, which can be attributed to their architectural design. The overall sound level of pink noise at a distance of 1 m is between 77.2 dB and 81.4 dB.

#### 4.1.3. Experiment Setup

In order to facilitate the positioning and subsequent referencing of the sources and receivers, taking advantage of the floor made up of 40 × 40 cm tiles, the entire test space is divided into grids, and a reference point is taken as the origin of coordinates. All receivers (microphones and sound level meters) will remain in a fixed position throughout the experiment. The sound sources (loudspeakers) are relocated, occupying a different position for each test. The loudspeakers are placed on the bases prepared for this purpose on one of their flat sides, and are adjusted to a height of 1.4 m. They are moved for each test, but their height remains constant.

**Figure 16:**
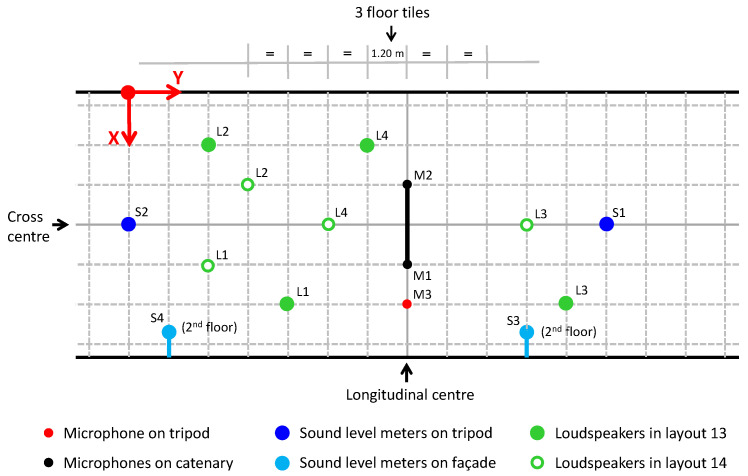
Plan view of the passageway with the arrangement of receivers and position of sources in two of the tests carried out. The origin of coordinates is shown at the top, with the *X*-axis transversal to the passage, the *Y*-axis longitudinal and the *Z*-axis perpendicular to the floor, with the origin of coordinates at the floor. A colour code and markers are used to identify the elements of the experiment.

Figure 16 shows a schematic diagram of the passageway, the arrangement of the receivers during the experiment and, as an example, the position of sources in two of the tests carried out. Table 1 provides a summary of the coordinates at which each receiver is situated in relation to the origin of coordinates. It is acknowledged that during the measurement period, any elements that could produce interferences or reflections, and thereby contaminate the measurement, should be excluded.

#### 4.1.4. Test Methodology and Results

A total of 17 tests are performed. In each test, the position of the sources or the number of active sources varies. Each test is conducted for a period of 15 s, during which the signal emitted by the sources is recorded with the receivers. Subsequently, the signal is processed on a second-by-second basis, resulting in the generation of an equivalent continuous spectrum and an overall sound level at each receiver for this measurement time interval. At the beginning and end of the experiment, measurements of wind speed, temperature and relative humidity are taken to check that these parameters remain within the limits set by the manufacturers of the receiving devices.

Each of the tests performed during the experiment are solved with the numerical model following the procedure proposed in Section 2.4, using the spectra represented in Figure 14b and the half-space solution (7) to establish the reference that characterises the noise sources used. The results presented comprise the spectrum and the overall sound pressure level at each receiver in the experiment. As the noise sources are in a fixed position in each arrangement and to take into account the variability of the acoustic field due to the Street Canyon effect (Section 2.6), the model performs the calculation using spectral density, although the results are also displayed in one-third octave bands and at overall sound levels.

**Figure 17:**
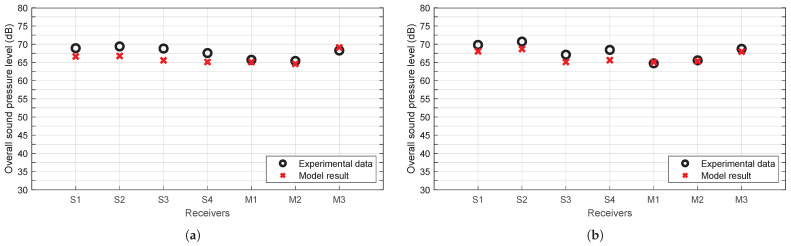
Overall sound pressure level, experimental data vs model results. (**a**) Results for layout 13. (**b**) Results for layout 14.

**Figure 18:**
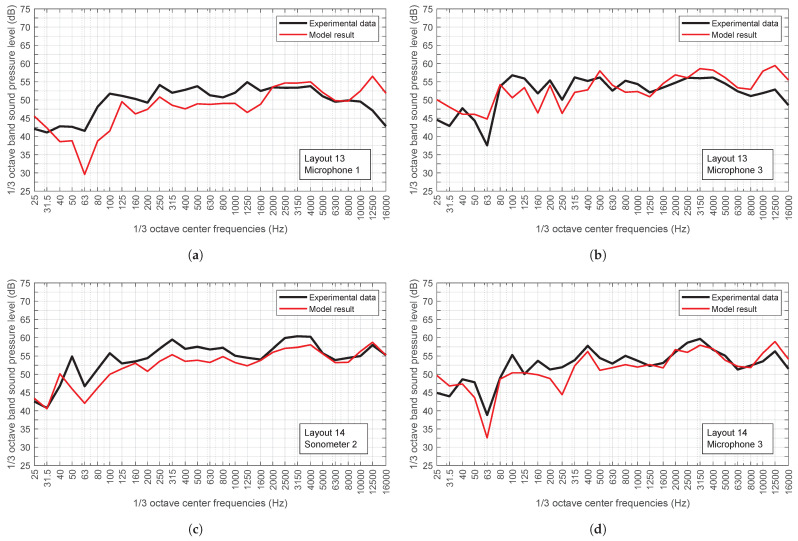
Spectra recorded by some of the receivers, experimental data vs model results. (**a**) Results for microphone 1 in layout 13. (**b**) Results for microphone 3 in layout 13. (**c**) Results for sonometer 2 in layout 14. (**d**) Results for microphone 3 in layout 14. All represented as $L_{Z}$ (dB). A logarithmic scale was employed for the abscissa axis in all graphs.

Figure 17 shows, for the sake of brevity, the overall sound pressure level recorded by each receiver used in both layouts shown in Figure 16, compared with the results returned by the model for the same receiver positions. Also shown in Figure 18 is a comparison between the spectrum recorded by two of the receivers in the same layouts and the spectra returned by the model in these positions.

It can be observed that both the spectra and the overall sound levels provided by the model are in close alignment with those recorded during the experiment. However, it should be noted that the largest discrepancies are those obtained with commercial sound level meters. This is most likely attributed to the fact that these devices apply a specific frequency and time weighting to the recorded signal, which is not accounted for in the model. In the experiments conducted as part of the present study, the sound level meters were configured to utilise a Fast time weighting.

**Figure 19:**
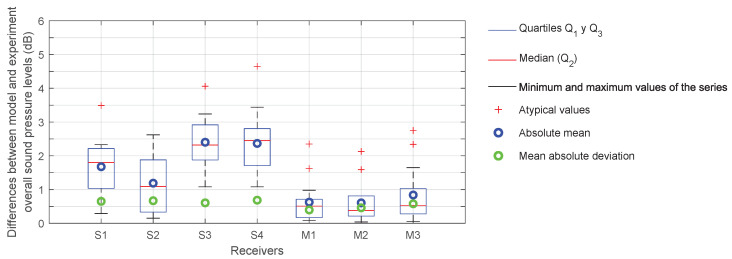
Box plots and mean absolute deviation of the differences between experiment and Street Canyon model for all 17 tests performed.

In addition, the discrepancies between the experimental data and the model outcomes for the overall sound levels are calculated and illustrated in box plots (Figure 19). The absolute differences obtained for each receiver in all the tests are employed in the generation of these box plots so that the analysis is performed per receiver. Also, the mean absolute deviation between tests for these differences at each receptor is included in the same graph.

It can be observed that the discrepancies between the model outcomes and the experimental data are not significant. This suggests that the proposed Street Canyon model is capable of accurately reproducing the behaviour of the acoustic energy present. The impact of façade reflections is estimated to be approximately 6 to 8 dB on average, contingent on the position of the receiver and the test in question. In order to quantify this effect, a propagation model was employed, which utilises the half-space solution (without façades). Thus, it can be concluded that the model accurately represents the effect of façades on sound propagation and that, as expected, the influence of façades is significant in the experimental data obtained in these tests.

### 4.2. Validation of the Model in Real Situations

In this experiment, the ability and reliability of the complete procedure to reproduce the intended problem will be evaluated. Already in a real context with anthropogenic noise, the suitability of this proposal and all its elements to predict noise levels from the number of people on the street, their distribution and the surrounding urban geometry will be validated.

For this purpose, several measurement campaigns were carried out in two streets of the city of Las Palmas de Gran Canaria (Canary Islands, Spain). This city is located in the northeastern quadrant of the island of Gran Canaria and covers an area of just over 100 ${\mathrm{km}}^{2}$. With a population of over 380,000, it is the most populous city in the Canary Islands and is the ninth most populous city in Spain as of 1 January 2024 [41,42]. Its main economic activity is tourism and all the activities related to it: accommodation, restaurants, shops, etc.

**Figure 20:**
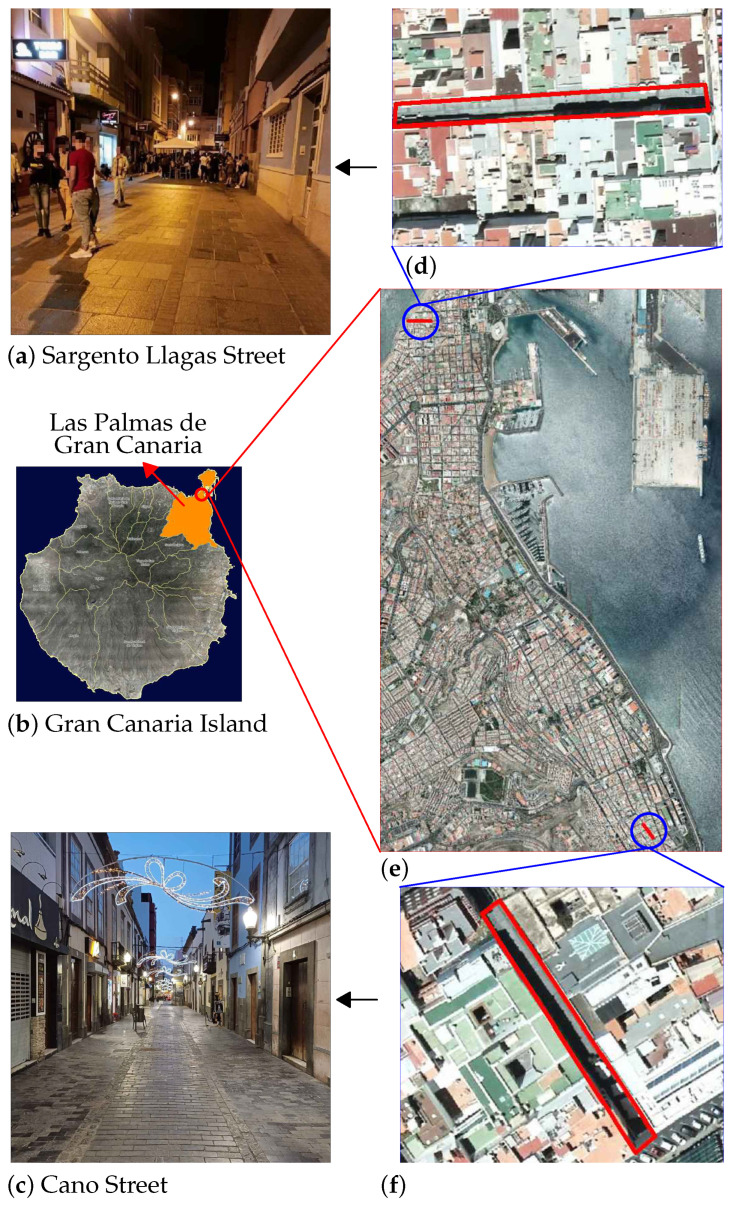
Situation map of measurement campaigns. (**a**,**c**) Photos taken by the authors. (**b**,**d**–**f**) Maps of the city of Las Palmas de Gran Canaria taken from [43].

The places where the measurement campaigns were carried out are Sargento Llagas Street and Cano Street (Figure 20). Both reproduce quite similarly the conditions of a Street Canyon model and are located in the two areas historically most associated with and affected by these types of activities. For this reason, both are pedestrian streets and are located in an area of the city where most of them are pedestrian streets. Thus, from the point of view of the intended experiment, the effect of traffic-related noise is very small. Both have multi-story buildings on either side along their entire length and are narrow in width, 7.5 m and 6.3 m, respectively. The campaigns cover different days of the week and different time slots, so there is considerable variability in noise and occupancy conditions.

#### 4.2.1. Devices and Materials

Camera: A GoPro Hero 8 Black (CA, USA) camera is utilised for the purpose of photographing the area under study, after which the number of people and their distribution are calculated. The camera is anchored using a Jaws model clamp and a flexible arm model FlexCamp 15 cm, both from the same manufacturer.

Receiver: A sound level meter Hottinger Brüel & Kjaer model 2250 (G4) is used. It is equipped with the weatherproof microphone kit employed for the sound level meters anchored to the façade in the validation with controlled artificial noise sources (Section 4.1). As in the experiment with controlled noise sources, prior to the start of the experiment, the sound level meters are calibrated using a sound level meter calibrator model 4231.

**Figure 21:**
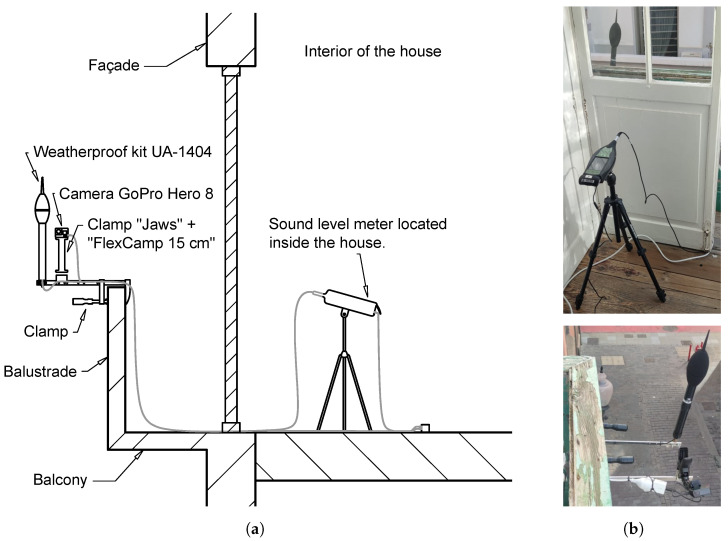
(**a**) Diagram of the balcony installation and assembly of the kit for measurement campaigns in urban areas. (**b**) Images of the installation in Cano Street.

Measurement kit: In order to facilitate the assembly of the measuring devices, portable equipment was developed. This equipment will take continuous static measurements and will integrate the sound level meter and the camera for taking images. A self-made fastening system is utilised to provide support for the constituent elements of the kit (See Figure 21).

#### 4.2.2. Experiment Setup

The measurement kit is anchored to the balconies and windows of neighbours residing in the street where the campaign is being conducted and who collaborate in its development, using the fixing system built. Figure 21 shows the final view of the measuring equipment once it was installed.

Three measuring devices were installed in these areas. Two of these are located in Sargento Llagas Street, and the third is situated in Cano Street. The height of the installation sites varies as follows:

- Receiver R1: Sargento Llagas Street (first floor, 5.45 m from ground);
- Receiver R2: Sargento Llagas Street (fourth floor, 14.9 m from ground);
- Receiver R3: Cano Street (first floor, 7.1 m from ground).

#### 4.2.3. Test Methodology and Results

The sound level meter records continuously throughout the campaign, thereby obtaining an A-weighted equivalent continuous spectrum and overall spectral sound level records at one-minute intervals. Similarly, the camera is configured to record continuously throughout the campaign, also taking a picture every minute. During the measurement, both the camera and the sound level meter are synchronised, thus enabling the identification of noise levels in relation to occupancy levels.

It is evident that occupancy levels may fluctuate in that minute, a variation that may be pertinent in certain instances. However, a thorough examination of successive images was conducted, and it was determined that, in the majority of cases, this data is indicative of occupancy during that specific time period.

In Sargento Llagas Street, the campaigns were carried out during weekends and periods of celebratory events, with the objective of capitalising on the heightened occupancy levels and, therefore, the higher noise levels that these occasions typically entail. In Cano Street, the campaigns were conducted over extended periods, encompassing all days of the week and both festive and non-festive periods. This approach enables the analysis of the reliability of the model for both low- and high-crowd-density scenarios. The duration of these campaigns ranges from three days (a weekend) to several weeks. For instance, the campaign in Sargento Llagas Street was conducted during February 2023. In Cano Street, the campaign was conducted between December 2023 and March 2024.

**Figure 22:**
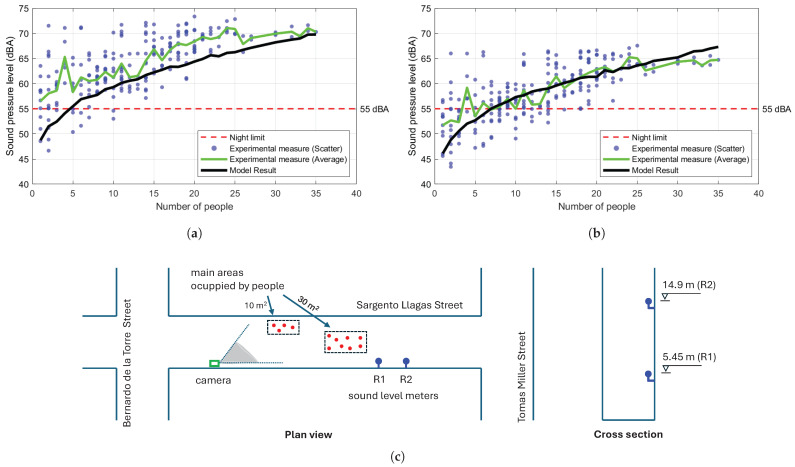
Results of the model and measures of the campaign located in Sargento Llagas Street. Records from the night period of the 10th, 11th, 17th, 18th, 19th and 20th of February 2023. (**a**) Receiver 1, located on the first floor. (**b**) Receiver 2, located on the fourth floor. (**c**) Sketch showing the positions of the measuring devices and the area analysed; plan and section views are out of scale, only for descriptive purposes of the problem and experimental setup.

**Figure 23:**
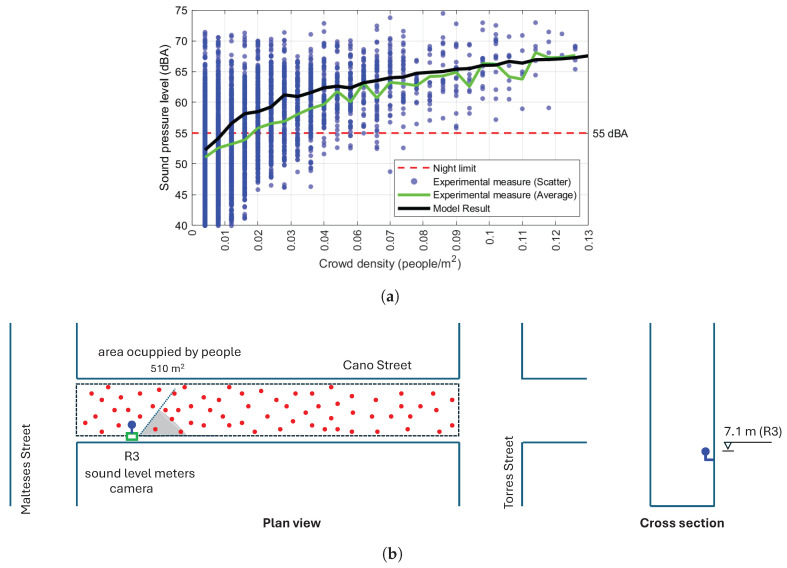
Results of the model and measures of the campaign located in Cano Street. Records of the night period from the 22nd to the 26th of December 2023, from the 8th to the 20th of February 2024 and from the 22nd to the 29th of March 2024. (**a**) Receiver 3, located on the first floor. (**b**) Sketch showing the positions of the measuring devices and the area analysed; plan and section views are out of scale, only for descriptive purposes of the problem and experimental setup.

Experimental and model-predicted sound levels in both places are presented in Figure 22 and Figure 23. The abscissa axis shows the crowd density or number of people, as ascertained from the images captured by the camera (the number of sources and area photographed). The ordinate axis illustrates the equivalent continuous sound pressure level (dBA) recorded experimentally or the sound pressure level computed with the numerical model for each occupancy data during the analysed period. All the records presented were taken during the time period corresponding to “night” (23:00 to 07:00) as it appears in the Spanish legislation related to this problem (RD 1367/2007, [44]). The noise limit level specified in that standard for that time period (55 dBA) is also indicated.

The average value of the equivalent continuous sound level measured experimentally for each occupancy data during that period is also represented. To obtain this average value, outliers are discarded, and the arithmetic mean of the remaining values is taken. Any value that falls outside the interquartile range, as measured from the first or third quartile, is also excluded.

The sound levels predicted by the model are presented with the same range of crowd densities. In each case, the corresponding number of sources is randomly distributed over the reference zones and 30 calculation iterations are performed also incorporating a random phase shift between sources. The arithmetic mean of the results obtained for each crowd density in these iterations is plotted. Recall that this calculation process is performed as explained in Section 2.4, and as there is indicated, the sound emission intensity of each source at 1 m is established from the crowd density in each of the occupied zones according to Equation (10). The model performs a broadband analysis using the pink noise spectrum in one-third octave bands so that the calculation process even for high occupancy levels is completed in a few seconds.

In consideration of the characteristics of the areas of analysis and the nature of the problem, it can be deduced that there are undoubtedly other sources of noise in addition to that produced by people. However, upon consideration of the data collected and the correlation between noise levels and occupancy rates, it can be substantiated that the noise produced by crowds constitutes the predominant source of impact in the cases examined. Consequently, it can be concluded that the designated areas are particularly well suited to the specific problem addressed in this study.

The results for Sargento Llagas Street are shown in Figure 22 for receivers R1 (Figure 22a) and R2 (Figure 22b). The dots show the distribution of experimentally measured sound levels during the indicated period, while the continuous lines represent the averages of such data and the average results obtained from the numerical model for each occupancy level. Taking into account that the occupancy density in each of the areas may be different, the total number of people captured by the camera is used as the variable on the abscissa axis.

A sketch showing the positions of the sound level meters, the camera and the occupied areas is also included (Figure 22c). In this case, the distribution and success of the leisure establishments means that the people occupying the street are mainly concentrated in two specific zones located close to them. The camera is placed at one end of the grid, on a second-floor balcony, and oriented in the direction that allows capturing both zones. As previously indicated, the sound level meters are outside two apartments close to the occupied zones and located at different heights (R1 and R2).

With the same pattern, Figure 23 shows the results for Cano Street. In this case, only one house was available for data collection, where a camera and a sound level meter were installed (Figure 21). The location allows the camera a very good perspective of the street, and the visibility conditions are adequate. In this case, the number, type and location of establishments and shops means that the distribution of people is uniform along the entire stretch of street analysed. For this reason, the crowd density on the abscissa axis is used in this graph, which is the most appropriate and informative parameter in this case.

The conditions of visibility and the location of the occupied areas, the levels of concentration of people in them, as well as the location of the cameras resulted in the quality of the images taken in both streets being very different. In Sargento Llagas Street, the general quality of the images was poor, and YOLOv8 had many difficulties processing them and correctly counting the number of people present at each moment. Post-processing was necessary to select only those images that were correctly treated. In Cano Street, however, the software was able to work automatically, reporting occupancy data for all the moments in which sound level records were taken. This is the reason why the number of points in the distribution is very different in both streets when the time periods in which data were taken were similar.

Despite this, in both experiments and for all receivers, the average sound levels reported by the model are very similar to those measured experimentally for medium and high occupancy levels in the range in which data are available. It should also be noted that the results for low occupancy levels are not representative, and it is necessary to extend the validation of the model for higher occupancy levels. However, it can be concluded that this procedure, with all its assumptions, simplifications and uncertainties, is able to predict in real time with acceptable efficiency and accuracy the anthropogenic sound levels from a few occupancy distribution data processed from images.

#### 4.2.4. Applicability as a Real-Time Noise Monitoring System

For the applications described in the introduction, it is not necessary to provide a second-by-second noise estimation. In the present context, it seems reasonable to accept “real-time” as a system that provides a noise estimation every 1, 2, 5, 10 or even 15 min, depending on the monitoring requirements.

Although the proposed methodology has not yet been implemented as an integrated real-time noise monitoring system, the different implemented components exhibit reasonable computational costs. In particular, when using a simple laptop (Intel (R) Core (TM) i7-12700H 2.70 GHz, 16 GB RAM):

- The number of people in the streets is estimated by YOLOv8 in tenths of a second. This may be accelerated further (to even a few miliseconds) if a GPU is used.
- The mathematical model for estimating the sound pressure level takes a variable amount of time depending on the number of people detected (sound sources), the amount of people distribution iterations and the number of receivers. In the cases studied in this paper, it took up to 30 s.

Therefore, a simple extrapolation of these results shows that it may be possible to use 15 cameras located around a given city zone and provide a 15 min noise estimation using a simple desktop computer. Even at this early stage, the scalability of the methodology is promising.

## 5. Conclusions and Future Research Directions

This paper proposes a strategy designed to predict the level of noise produced by crowds of people that is not based on direct measurement using sound level meters, but on an indirect procedure that makes use of computer vision and artificial intelligence techniques. This proposal is based on the development and verification of two interconnected tools: (1) a procedure that uses images to determine the density and distribution of the crowd in pedestrian streets, and (2) a numerical model that uses this information and the urban geometry to efficiently calculate the noise level at any point in the analysis area. If this were possible with an infrastructure that in many cases already exists for another use (security systems and cameras), it would also be possible to obtain an estimate of the level of noise pollution in a given area of the city. In particular, it presents the basis of this strategy and an initial mathematical model to address the problem in a simplified real situation. All the essential aspects of the procedure are described, as well as an experimental validation at two levels that allows us to limit some of the uncertainties inherent to this phenomenon: (1) a laboratory validation using noise sources with controlled emission spectra, and (2) validation in a real situation of anthropogenic noise in two streets of the city of Las Palmas de Gran Canaria, Canary Islands, Spain. The main conclusions that the reader can draw from this work at this stage are the following: (1) the proposed procedure is simple and the tools and models are easily accessible, and (2) although the results are very preliminary, the experimental validation allows the conclusion to be reached that this strategy is able to satisfy the proposed objective with a more than acceptable accuracy in a situation where the main source of noise is of anthropogenic origin. It is true that it is necessary to extend the measurement campaign to other streets and at other times in order to validate the behaviour of the model (image processing and noise evaluation) at higher occupancy levels.

As for future research directions, these are mainly related to the development and evolution of the mathematical model of acoustic propagation:

1. Incorporate boundary conditions into the Street Canyon model to simulate the absorptive capacity of the façades or the diffuse nature of the acoustic field in their vicinity resulting from successive diffractions/reflections produced by the façade elements [12].
2. Represent the acoustic field infiltrating the interior of homes through open (or half-open) windows in the façades. In this case, it is proposed to generalise the presented solution of the Street Canyon to the interior problem and to couple both regions in a 3D code based on the Boundary Element Method (BEM) previously developed by some of the authors (MultiFEBE, [45]).
3. Extend the numerical model to represent a significant part of the affected urban grid, including all streets (crowd-occupied or not) and their actual geometry. Performing this task by proposing a completely realistic frequency domain 3D numerical model is virtually unfeasible (the frequency range analysed, the speed of sound propagation in air and the dimensions of the area to be treated). Using the BEM as a numerical strategy, the authors propose to develop a dimensionally simpler model using 2.5D techniques that reduce the problem to two dimensions without losing its 3D character (see, e.g., [46]).
4. In line with previous work by the authors [47], investigating the effectiveness of acoustic screens for this problem, from simple temporary and mobile solutions (see, e.g., [48]) to elements permanently installed on windows and façades of affected buildings [49].

All these tasks include the experimental validation of the resulting models as they are developed. From them, it will be possible to progressively adjust an increasingly accurate tool that permits the determination, in real time, of noise pollution levels from image processing alone. In addition to this use as a tool for noise level assessment in the direction initially proposed in this work, it is also intended that these numerical models, by themselves, will be useful as a versatile and general analysis software for the study of the problem. Therefore, it is also the aim of this work to make all the results and models available in open access for interested researchers.

## Figures and Tables

**Table 1:** Coordinates of the receivers, referenced to the origin of coordinates considered in Figure 16.

Receiver	Coordinates		
	X	Y	Z
Sound level meter S1	4.15	14.40	1.37
Sound level meter S2	4.15	0.00	1.37
Sound level meter S3	7.30	12.00	5.50
Sound level meter S4	7.30	1.20	5.50
Microphone M1	5.35	8.40	8.55
Microphone M2	2.95	8.40	8.55
Microphone M3	6.55	8.40	1.37

Z height above the ground. Coordinates in metres.

## Data Availability

The original data presented in the study are openly available in Zenodo at https://doi.org/10.5281/zenodo.15186086.
